# Endoscopic Submucosal Resection With a Ligation Device for Pediatric Rectal Neuroendocrine Tumor

**DOI:** 10.14309/crj.0000000000001669

**Published:** 2025-04-10

**Authors:** Ryosuke Miyamoto, Hitoshi Honma, Yu Masuda, Tomoya Sugiyama, Satoshi Ono, Akihisa Okumura

**Affiliations:** 1 Department of Pediatrics, Aichi Medical University School of Medicine, Nagakute, Aichi, Japan; 2 Department of Gastroenterology, Aichi Medical University School of Medicine, Nagakute, Aichi, Japan

**Keywords:** rectal neuroendocrine tumor, ESMR-L

## Abstract

A 13-year-old boy with a 3-month history of diarrhea was successfully treated for a rectal neuroendocrine tumor (NET) using endoscopic submucosal resection with ligation (ESMR-L). Rectoscopy revealed a submucosal tumor in the lower rectum, diagnosed as a rectal NET upon pathological examination. ESMR-L resulted in complete tumor resection without complications. Pathological examination confirmed a 6-mm low-grade NET with no vascular invasion or positive tumor margins. The patient was discharged without complications. This case study indicates that ESMR-L can be applied to rectal NET in children, necessitating further investigation on its efficacy and safety in more patients.

## INTRODUCTION

Neuroendocrine tumors (NETs) that originate from neuroendocrine cells—the cells with both neuronal and endocrine characteristics—are relatively rare yet predominant in individuals aged 50–60 age.^1,2^ More than half of NETs occur in the gastrointestinal tract, most commonly in the small intestine (45%), rectum (20%), appendix (16%), colon (11%), and stomach (7%).^3^ NETs can be classified into nonfunctional or functional. Approximately 15% of patients present with carcinoid syndrome characterized by facial flushing, diarrhea, and bronchospasm, whereas others may present with nonspecific symptoms such as weight loss, bleeding, and abdominal pain or are asymptomatic.^4^

Endoscopic submucosal resection with a ligation device (ESMR-L) is a modified endoscopic mucosal resection (EMR) technique, wherein an endoscope is fitted with a ligation band and the lesion is aspirated while band snaring is performed using radiofrequency.^5,6^ In this study, we report the successful treatment of a pediatric patient with rectal NET using ESMR-L with no further complications.

## CASE REPORT

A previously healthy 13-year-old boy with difficulty attending school due to a 3-month history of diarrhea was diagnosed with irritable bowel syndrome at a local hospital. The patient was prescribed probiotic medications; however, they proved ineffective. Subsequently, rectoscopy was performed to detect ulcerative colitis. A 10-mm submucosal tumor in the lower rectum was diagnosed as a G1 rectal NET on pathological examination. Although the diarrhea resolved spontaneously, the patient was admitted to our hospital for treatment. On presentation, his abdomen was flat and soft without tenderness, with no palpable mass during rectal examination. Furthermore, no facial flushing or redness of the pharynx or tongue were observed. Laboratory examination revealed Na: 141 mEq/L, K: 4.3 mEq/L, Cl: 106 mEq/L, corrected Ca: 9.5 mg/dL, insulin: 4.7 μU/mL, and intact-parathyroid hormone: 34 pg/mL. Abdominal magnetic resonance imaging revealed no notable intrahepatic metastases. The tumor was considered a nonfunctional NET as normal values in the electrolyte or intact parathyroid hormone were observed.

**Figure 1.**
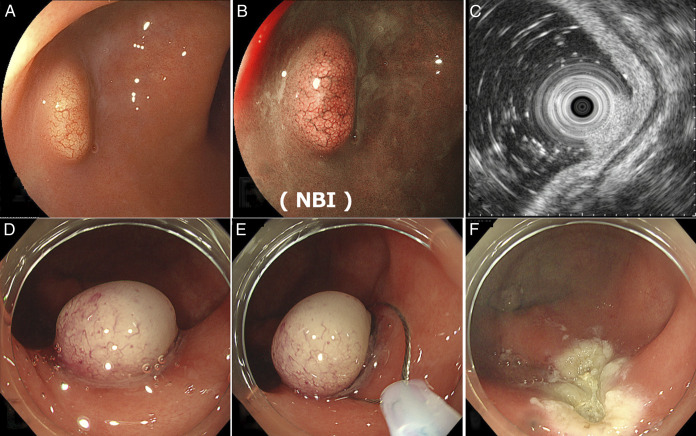
The resection procedure. (A and B) Tumor identification. (C) Endoscopic ultrasound revealed tumor depth in the submucosa. (D) The tumor root was ligated with a ring. (E) The tumor was resected with a high-frequency snare. (F) Slight bleeding was observed from the resection site. NBI, narrow-band imaging.

**Figure 2.**
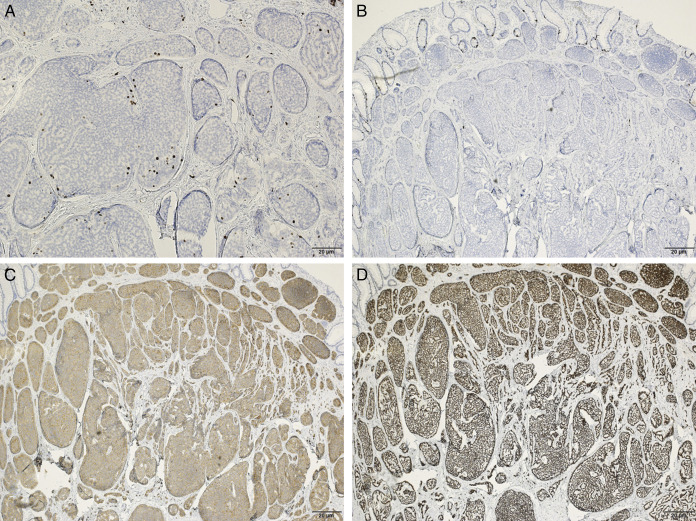
The pathological results of the patient. Immunohistochemical studies revealed (A) (200×) a Ki-67 index <2% and (B) (×40) negative for chromogranin A. (C and D) (×40) positive results were obtained for synaptophysin and insulinoma-associated protein 1 immunostaining.

We selected ESMR-L for treatment because of its higher tumor resection rate than EMR and simpler technique than endoscopic submucosal dissection (ESD). Before the procedure, the entire colon was observed under sedation using a PCF-H290ZI (Olympus Medical Systems Co., Tokyo, Japan). The endoscope was fitted with a transparent cap to increase visibility and facilitate the procedure. The tumor was confirmed to be solitary in the lower rectum (Figure 1). Endoscopic ultrasound revealed that the tumor was confined to the submucosa (Figure 1). Subsequently, 1 mL of a solution containing half hyaluronic acid (Muco-up; Johnson & Johnson K.K. Medical Co.) and half saline was injected locally into the tumor submucosa to lift up, which was then ligated with an endoscopic band ligation device (SB-KAWASUMI Laboratories, Inc., Kawasaki, Japan) to interrupt blood flow and excised using a high-frequency snare ENDO CUT Q by effect 3, cut duration 1, cut interval 4 (Erbe Elektromedizin GmbH, Germany) (Figure 1). The procedure required approximately 10 minutes to remove the tumor. No bleeding was observed in the treated area (Figure 1). The pathological findings were as follows: tumor diameter: 6 mm, Ki-67 immunostaining, which quantifies cell proliferation, index: <2%, and tumor classification: low-grade. Furthermore, chromogranin A, the biomarker of NETs, immunostaining was negative, whereas insulinoma-associated protein 1, involved in neuroendocrine cell differentiation, and synaptophysin, the biomarker, immunostaining was both positive (Figure 2). Vertical and horizontal margins were negative for tumors, and Victoria Blue staining and D2-40 immunostaining showed no vascular invasion. The tumor was completely resected. The patient was discharged the following day with no notable abdominal pain, fever, or other complications.

## DISCUSSION

A rectal NET of a 13-year-old boy was completely resected by ESMR-L, with no further complications. Reports of NETs in children are limited, as pediatric NETs are rare. The incidence of NETs in adults is 7/100,000 in the United States and 3.5/100,000 in Japan.^7,8^ Notably, the increase in screening colonoscopies has resulted in the increased incidence of NETs in recent years.^9^ Meanwhile, NETs are reported more frequently in the lungs, breasts, and appendices of children and young adults, with a very low incidence of 0.4–0.6 per million.^10^ This may partly be attributed to the difficulty of performing screening colonoscopies in children. NETs are not always easily detectable. Hormone-producing tumors or functional NETs account for approximately 15% of all gastrointestinal NETs.^4^ The incidence of functional NETs is even lower, particularly in children.^11^ Asymptomatic NETs are often detected incidentally during screening colonoscopy.^12^ NETs have been identified incidentally in the appendix of children during surgical treatment for suspected appendicitis.^13^ These facts suggest that the detection of NETs is challenging due to the absence of clinical symptoms that could serve as diagnostic clues. In our patient, diarrhea prompted the examination, leading to the early detection of the tumor, as it was located in the lower rectum, where it could be easily located.

We selected ESMR-L for treatment of NET in our patient. The European Neuroendocrine Tumor Society guidelines state that tumors <10 mm in diameter rarely indicate malignancy and do not exhibit a high risk of metastasis.^14^ Therefore, the tumor could be resected endoscopically. Various techniques, including endoscopic polypectomy, EMR, modified EMR, and ESD, are used for endoscopic treatment of rectal NETs. Among these techniques, ESMR-L exhibits a higher tumor resection rate than conventional EMR and is comparable to ESD in the vertical margin positivity rate, with shorter (<10 minutes) procedural time. In particular, ESMR-L requires 6–7 minutes, whereas ESD requires 24–43 minutes.^12,15^ The procedure in the present case lasted approximately 10 minutes, probably because of the involvement of a pediatrician with limited experience. Notably, reports of ESMR-L in children are limited, and we could find only one biopsy report of the diagnosis of Hirschsprung's disease.^16^ Nevertheless, we believe that the benefits of ESMR-L outweigh its disadvantages, and ESMR-L would be the most appropriate treatment option, particularly because the gastrointestinal wall in children is thinner than that in adults and ESD may cause perforation. The tumor was completely resected without complications.

In conclusion, we successfully treated a rectal NET in a boy using ESMR-L and achieved complete tumor resection. ESMR-L was completed within 10 minutes with no complications. ESMR-L represents an excellent treatment option for rectal NET, even in children. However, its efficacy and safety should be verified in more patients.

## DISCLOSURES

Author contributions: All coauthors have contributed significantly to the study and agree with the manuscript's content. All authors contributed to the conception and design of this study. Material preparation and data collection were performed by R. Miyamoto, H. Honma, Y. Masuda, T. Sugiyama, S. Ono, A. Okumura. The first draft of the manuscript was written by R. Miyamoto, and coauthor commented on previous versions of the manuscript. All the authors have read and approved the final version of the manuscript. R. Miyamoto is the article guarantor.

Financial disclosure: None to report.

Informed consent was obtained for this case report.
